# Comparative Study on Phytochemical Profiles and Antioxidant Capacities of Chestnuts Produced in Different Geographic Area in China

**DOI:** 10.3390/antiox9030190

**Published:** 2020-02-25

**Authors:** Ziyun Xu, Maninder Meenu, Pengyu Chen, Baojun Xu

**Affiliations:** 1 Food Science and Technology Programme, Beijing Normal University-Hong Kong Baptist University United International College, Zhuhai 519087, China; ziyun.xu@mail.mcgill.ca (Z.X.); meenu_maninder@yahoo.com (M.M.); l630013005@mail.uic.edu.hk (P.C.); 2 Department of Food Science and Agricultural Chemistry, McGill University, Quebec, QC H9X 3V9, Canada

**Keywords:** chestnuts, *Castanea mollissima*, phenolic properties, antioxidant capacities

## Abstract

This study aimed to systematically assess the phenolic profiles and antioxidant capacities of 21 chestnut samples collected from six geographical areas of China. All these samples exhibit significant differences (*p* < 0.05) in total phenolic contents (TPC), total flavonoids content (TFC), condensed tannin content (CTC) and antioxidant capacities assessed by DPPH free radical scavenging capacity (DPPH), ABTS free radical scavenging capacities (ABTS), ferric reducing antioxidant power (FRAP), and 14 free phenolic acids. Chestnuts collected from Fuzhou, Jiangxi (East China) exhibited the maximum values for TPC (2.35 mg GAE/g), CTC (13.52 mg CAE/g), DPPH (16.74 μmol TE/g), ABTS (24.83 μmol TE/g), FRAP assays (3.20 mmol FE/100 g), and total free phenolic acids (314.87 µg/g). Vanillin and gallic acids were found to be the most abundant free phenolic compounds among other 14 phenolic compounds detected by HPLC. Overall, the samples from South China revealed maximum mean values for TPC, CTC, DPPH, and ABTS assays. Among the three chestnut varieties, *Banli* presented prominent mean values for all the assays. These finding will be beneficial for production of novel functional food and developing high-quality chestnut varieties.

## 1. Introduction

Chestnuts (*Castanea* spp.), belonging to family Fagaceae, are extensively cultivated in Asian countries. China is the largest producer of chestnut followed by Bolivia, Turkey, Korea, and Italy [1]. Chestnuts were mainly produced from four economically important species, namely *Castanea mollissima* (Chinese chestnut), *C. crenata* (Japanese chestnut), *C. dentate* (American chestnut) and *C. sativa* (European chestnut) [2]. Chinese chestnut variety is preferred by its high yielding and easy cultivation [3]. It is a rich source of carbohydrates, fiber and minerals [4]. Fresh Chinese chestnut fruits exhibit significant amount of water (52.0%), carbohydrates (42.2%), proteins (4.2%), and lipids (0.7%) [5]. According to a prehistoric encyclopedia of China Compendium of Materia Medica (Ben Cao Gang Mu) from Ming Dynasty (A.D. 1590), Chinese chestnuts improve kidney functioning [6]. Thus, chestnuts are popular among the Chinese population from ancient times due to its nutritional value as well as the health benefits attributed to the presence of various antioxidant compounds [7]. Antioxidants, such as phenolic acids and their derivatives, are the group of naturally occurring functional substances in plant-based foods, especially in fruits, vegetables, and nuts. Chestnuts presented abundant antioxidant content (4.7 mmol Fe^2+^/100 g) compared to many legumes (0.11–1.97 mmol Fe^2+^/100 g), fruits (0.4–2.4 mmol Fe^2+^/100 g), and grain products (0.5–1.3 mmol Fe^2+^/100 g) [8]. Phenolic compounds present in chestnuts are responsible for free radical scavenging properties that in turn exhibit protective effects against coronary heart disease, cancer, neurodegenerative diseases and osteoporosis [9]. As a typical group of phenolic compounds, phenolic acids accounted for about 1/3 of phenolic compounds in plant-derived food, and most of them were derivatives of benzoic acid and cinnamic acid, existed in form of both free and bound [10].

Based on climatic characteristics of China, chestnut is mainly produced in five different geographical areas, namely North China, East China, Central China, South China, and Southwest China. Chinese chestnuts (*C. mollissima*) are classified into three subgroups, i.e., *Banli*, *Youli,* and *Maoli* based on the different morphological features. Among these varieties, *Maoli* is the smallest in size and contains a comparatively higher level of sugar and glutinous starch content [11]. Banli is the most common variety of chestnut and the fruit is flattened on one or two sides. Youli variety of chestnut exhibits round shape, darker color and lustrous outer shell.

Various researchers have studied the phenolic profiles and antioxidant activities of chestnuts collected from various geographical regions and also explored the impact of different processing techniques on phenolic content and antioxidant capacities of chestnuts [9,12,13,14]. A previous study reported the variation in phenolic content and flavonoids content of Chinese chestnut collected from North China to South region of China [15].

Although many researchers have investigated the antioxidant activities of various chestnut species from several geographical regions, phenolic profile in terms of total phenolic contents (TPC), total flavonoids content (TFC), condensed tannin contents (CTC), and antioxidant properties of three sub-varieties (*Banli, Youli*, and *Maoli*) of Chinese chestnuts (*C. mollissima*) collected from different geographic regions are still unexplored. Besides, phenolic profile in terms of 14 free phenolic acids of Chinese chestnuts were also unknown. Thus, the present study was carried out with an aim to systematically assess the phytochemical profiles as well as antioxidant capacities of twenty one raw chestnut fruits grown in five geographic areas of China.

## 2. Materials and Methods

### 2.1. Chestnuts Produced in Different Parts of China

The chestnut samples were collected from five different geographic areas in China in 2016. All the chestnut samples were identified as *Castanea mollissima* Blume by Professor Jingzheng Zhang from Chestnut Research Center, Hebei Normal University of Science and Technology, Hebei, China. All the collected samples were further classified as *Banli*, *Maoli,* and *Youli* based on their morphological features. Soil source for all chestnuts was sandy loam soil (pH 5.5–6.5). Harvested chestnuts were stored in specialized refrigerator (2–6 °C). The information regarding the common name, size, the specific growing area, average temperature, and monthly sunlight duration from April to September [16] is summarized in Table 1. The Supplemental Figure S1. is presenting the sampling geographical regions in China. The Supplemental Figure S2. is presenting the morphological appearance of twenty-one chestnut samples explored in this study.

### 2.2. Chemicals and Reagents

Folin-Ciocalteu reagent and 2, 2′-azino-bis (3-ethylbenzothiazoline-6-sulfonic acid) (ABTS) were purchased from Shanghai Yuanye Biological Technology Co., Ltd. (Shanghai, China). (+)-Catechin, 2,4, 6-tri(2-pyridyl)-s-triazine (TPTZ), and 2,2-diphenyl-1-picrylhydrazyl (DPPH) were obtained from Sigma-Aldrich (Shanghai, China). Absolute ethanol, 6-hydroxy-2,5,7,8-tetramethylchromane-2- carboxylic acid (Trolox), acetone, and methanol were provided by Tianjin Fuyu Fine Chemical Co., Ltd. (Tianjin, China). Trifluroacetic acid (TFA), butylated hydroxytoluene (BHT), and methanol (HPLC grade) were purchased from Sigma-Aldrich Co., Ltd. (Shanghai, China). All chemicals employed in this study were of analytical grade.

### 2.3. Sample Preparation

Chestnut samples were peeled with a chestnut peeler (550 W, Kenong Technology Co., Ltd., Jiangsu, China) and stored overnight at −80 °C. Samples were then freeze-dried using freeze-dryer (Freezone Benchtop, Labconco Corporation, Kansas City, MO, USA) and ground into fine flours. The percentage yield of dried chestnut flours was calculated by dividing dried chestnut flour weight by the weight of the fresh chestnut fruit.

### 2.4. Determination of Moisture Content and Color Attributes

The moisture content of dried chestnut flours was determined by the fast water content analyzer (MA150, Sartorius Corporation, Goettingen, Germany). Colorimeter (CR-410, Konica Minolta, Japan) was used to measure the color of all the chestnut samples. The color was expressed based on a three-axis color system L*a*b*; here L* denotes lightness, a* represents red (+) or green (−), and b* represents yellow (+) or blue (−). The colorimeter was calibrated with a standard white background plate before measurement.

### 2.5. Extraction of Total Phenolics from Chestnut Samples

For the extraction of phenolics, 0.5 g of chestnut powder was extracted with 5 mL of extraction solvent (acetone/water/acetic acid: 70:29.5:0.5, *v*/*v*/*v*) according to the previously mentioned procedure [17].

### 2.6. Determination of Total Phenolic Content (TPC)

TPC was determined by employing Folin–Ciocalteu assay as described by Xu and Chang [17]. Gallic acid was used as an external standard. The absorbance of the reaction mixture was measured at 765 nm using UV-Vis Spectrophotometer (UT-1901). TPC values of chestnut samples were expressed as milligram gallic acid equivalents per gram freeze-dried sample (mg GAE/g).

### 2.7. Determination of Total Flavonoid Content (TFC)

The TFC of chestnut samples was determined using colorimetric assay as described previously [17]. (+)-Catechin was used as an external standard and the absorbance of the reaction mixture was determined at 510 nm using UV-Visible spectrophotometer. TFC values were expressed as milligram (+)-catechin equivalents per gram of freeze-dried sample (mg CAE/g).

### 2.8. Determination of Condensed Tannin Content (CTC)

The CTC was determined according to the method described by Xu and Chang [17] with slight modifications. Catechin was used as an external standard and the absorbance of the resultant reaction mixture was measured at 500 nm using a UV-visible spectrophotometer. The CTC of chestnut samples was expressed as (+)-catechin equivalents per gram of freeze-dried sample (mg CAE/g).

### 2.9. DPPH Free Radical Scavenging Activity (DPPH) Assay

DPPH values of samples were performed using Trolox as external standard according to the previously described procedure [17]. The absorbance of the resultant reaction mixture was measured using a UV-visible spectrophotometer at 517 nm against the ethanol blank. Results were expressed as micromole of Trolox equivalents per gram of freeze-dried samples (μmol TE/g).

### 2.10. Ferric-Reducing Antioxidant Power (FRAP) Assay

A colorimetric reaction assay was used to determine the FRAP values of chestnut samples according to the method described by Xu and Chang [17]. The absorbance of the reaction mixture was measured at 593 nm using a UV-visible spectrophotometer. The FRAP value was expressed as millimoles of Fe^2+^ equivalent (FE) per 100 g freeze-dried samples (mmol FE/100 g).

### 2.11. ABTS Free Radical Scavenging Assay

ABTS free radical scavenging capacities of samples were performed according to the method reported by Xu and Chang [17] with slight modifications. Trolox was used as an external standard and the absorbance of the reaction mixture was measured at 734 nm using a UV-visible spectrophotometer against the ethanol blank. Results were expressed as micromole of Trolox equivalents per gram of freeze-dried samples (μmol TE/g).

### 2.12. HPLC Analysis of Free Phenolic Acids

The free phenolic acid contents of chestnut samples were determined by HPLC (High Performance Liquid Chromatography) according to the method described by Xu and Chang [18]. Briefly, 0.5 g of ground sample was extracted with 5 mL extraction solvent (methanol/water/acidic acid/BHT = 85:15:0.5:0.2, *v*/*v*) twice. The mixture was filtered through Whatman no. 42 filter paper and the supernatant was evaporated at 40 °C until dryness. The residue was dissolved in 2.5 mL methanol (25%, *v*/*v*) and 20 μL of the extract was subjected to HPLC system (Waters, e2695 Separations Modulek, Milford, MA, USA) equipped with a photodiode array detector. A reverse phase Zorbax C18 column (5 μm, 250 × 4.6 mm) was employed at temperature of 40 °C. Mobile phase for analysis include solvent A (0.1% acetic acid in water) and solvent B (methanol). The flow rate was set at 0.7 mL/min and the working wavelength of the detector was set at 262 nm. The chromatograms of 14 phenolic acids were extracted at different maximum absorption wavelength from 210 nm to 320 nm. The contents of 14 free phenolic acids were expressed as microgram free phenolic acid per gram sample (g/g sample) on dry weight basis. The regressive equations and correlation coefficients for phenolic acid standards are provided in the Supplemental Table S1.

### 2.13. Statistical Analysis

All experiments were performed in triplicates and the data were expressed as mean ± standard deviation. The significant differences among mean values were analyzed using One-Way ANOVA. Duncan test was performed to determine the significant differences (*p* < 0.05) among the mean values of different samples. Statistical analysis was performed by using IBM SPSS Statistics version 22 (IBM Corporation, New York, USA).

## 3. Results

### 3.1. Yield of Chestnut Flours and Moisture Content

Among all the chestnut samples under investigation, the flour yield ranged from 39.11% in case of samples from Jixian, Tianjin (North China) to 61.17% in case of samples from Huairou (North China) as shown in Table 2. Moisture content values of all the chestnut samples exhibit a significant difference (*p* < 0.05) as shown in Table 2. Among all the samples under investigation, the highest moisture content was recorded as 13.14% in samples from Jixian, Tianjin, (North China) while the lowest value (5.02%) was found in samples collected from Anqing, Anhui, (East China).

### 3.2. Color Value

The color values of all the twenty-one chestnut samples are mentioned in Table 2. Among all the samples, the significant differences (*p* < 0.05) were observed in their color parameters L*a *b. The lightness value (L) of the samples were ranged from 85.77 in case of samples collected from Fuzhou, Jiangxi (East China) to 93.51 in chestnut samples from Wenzhou, Zhejiang (East China). The a* value was varied from −2.52 in samples from Yangjiang, Guangdong (South China) to 0.50 in case of samples collected from Fuzhou, Jiangxi (East China). The b* value was ranged from 7.63 in chestnut samples from Wenzhou, Zhejiang (East China) to 14.94 in case of samples belong to Yangjiang, Guangdong (South China).

### 3.3. Phenolic Profiles and Antioxidant Capacities of Chestnut Samples

The phenolic profiles in terms of TPC, TFC, and CTC values along with the antioxidant activity of all the chestnut samples as assessed by DPPH, FRAP, and ABTS assay are presented in Table 3. The samples collected from different geographical areas exhibited a wide range of variation among their TPC, TFC, CTC values, and antioxidant capacities.

Among all the chestnut samples, TPC values were ranged from 1.03 mg GAE/g in the samples collected from Zhaotong, Yunnan (Southwest China) to 2.35 mg GAE/g in case of samples belonging to Fuzhou, Jiangxi (East China). Overall, chestnut samples from South China exhibited higher mean TPC values (1.89 mg GAE/g) compared to the samples from other regions, whereas, the samples from Southwest China revealed minimum mean TPC values (1.41 mg GAE/g). It was also interesting to observe that the TPC values of chestnuts samples collected from Guilin (2.19 mg GAE/g) and Liuzhou (2.12 mg GAE/g) cities of Guangxi province were comparatively higher than samples collected from other regions in South China.

TFC values were ranged from 0.57 mg CAE/g in case of samples collected from Nanping, Fujian (East China) to 1.13 mg CAE/g in case of samples procured from Kunming, Yunnan (Southwest China). Overall, the samples from Southwest region of China demonstrated the highest mean value of TFC (0.88 mg CAE/g) and samples from North China exhibited least mean value for TPC (0.72 mg CAE/g) compared to samples from other regions. It was also observed that the TFC content of chestnut samples collected from different cities of the same province exhibit a significant difference (*p* < 0.05). In this study, TFC values of samples from Tangshan (0.78 mg CAE/g), Xingtai (0.68 mg CAE/g) and Qinhuangdao (0.84 mg CAE/g) cities of Hebei province presented significant differences (*p* < 0.05).

The chestnut samples procured from different geographical areas had also presented a wide range of variation in their CTC values. The highest CTC value (13.58 mg CAE/g) was observed in the case of samples collected from Liuzhou, Guangxi (South China). In general, chestnut samples from South China exhibit maximum mean value for CTC (9.41 mg CAE/g) and the samples from North China presented minimum mean value for CTC (7.24 mg CAE/g). Alike the TFC values, significant differences (*p* < 0.05) were also observed among the CTC values of samples collected from different regions of the same province.

DPPH values of chestnut samples under investigation were ranged from 7.08 μmol TE/g in case of samples procured from Zhaotong, Yunnan (Southwest China) to 16.74 μmol TE/g in case of samples from Fuzhou, Jiangxi (East China). Overall, among all the geographical regions, the highest mean DPPH value was exhibited by the samples from South China (11.76 μmol TE/g) and the lowest mean DPPH value was presented by the samples procured from Southwest China (8.30 μmol TE/g). Alike TFC and CTC values, DPPH values of chestnut samples collected from different cities of same province also exhibits a significant difference (*p* < 0.05) except the samples procured from Tangshan (10.18 μmol TE/g) and Xingtai (10.02 μmol TE/g) cities of Hebei province and the samples from Guilin (13.87 μmol TE/g) and Liuzhou (13.62 μmol TE/g) from the Guangxi Province.

The FRAP values of chestnut samples under investigation varied from 0.66 mmol FE/100 g in case of samples from Linyi, Shandong (East China) to 3.24 mmol FE/100 g in case of samples collected from Guilin, Guangxi (South China). Overall, the samples from South China presented higher mean FRAP value (2.37 mmol FE/100 g), whereas, the chestnut samples from Southwest China (0.99 mmol FE/100 g) exhibit lower mean FRAP values compared to the samples collected from other regions. It was also observed that the chestnut samples collected from different cities of the same province also exhibit significant differences (*p* < 0.05) in their FRAP values.

The ABTS values of chestnut samples collected from various regions of China also exhibit a wide range of variation. Among all the samples, chestnut procured from Zhaotong, Yunnan (Southwest China) exhibit the lowest ABTS value (6.53 μmol TE/g). Whereas, the samples from Fuzhou, Jiangxi (East China) exhibit higher ABTS value (24.83 μmol TE/g). While comparing the mean ABTS values presented by samples from five different geographical regions, it was observed that samples collected from East China exhibit the highest mean ABTS value (14.94 μmol TE/g) followed by the samples from Central China (14.69 μmol TE/g), South China (14.68 μmol TE/g), and North China (13.10 μmol TE/g). The lowest mean ABTS value (11.52 μmol TE/g) was presented by the samples collected from Southwest China.

### 3.4. Free Phenolic Acids Contents of Chestnut Samples

The chromatograms of 14 phenolic acids (including gallic acid, protocatechuic acid, 2,3,4-trihydroxybenzoic acid, protocatechualdehyde, *p*-hydroxybenzoic acid, gentisic acid, chlorogenic acid, vanillic and caffeic acid, syringic acid, vanillin, *p*-coumaric and syringaldehyde, ferulic acid, sinapic acid, and salicylic acid) determined by HPLC were shown in Supplemental Figure S3. Chemical structure of each phenolic compound was presented in Table 4. The contents of these phenolic acids in chestnut samples among different geographical areas were significantly different (*p* < 0.05), and values were summarized in Table 5.

Overall, the highest total phenolic content (314.87 µg/g) was observed in samples collected from Fuzhou, Jiangxi. Among five geographical regions, samples collected from East China were shown to have the highest mean value of total phenolic acids (211.37 µg/g), followed by South China (188.72 µg/g) and North China (183.85 µg/g).

In terms of individual phenolic compounds, vanillin was the most abundant phenolic compound and the contents were ranged from 21.23 µg/g in the sample collected from Xinyang, Henan to 99.33 µg/g in the sample collected from Haikou, Hainan. Overall, samples from North China contained the highest mean value of vanillin among all five different regions, whereas samples from Central China contained the lowest amount. Besides, although high amount of vanillin was found in North China region, no significant differences (*p* < 0.05) were found in chestnut samples between different cities. However, significant differences of vanillin content were found in samples collected from cities in South China.

Gallic acid was found to be the second dominant phenolic compound in chestnut samples. Contents of gallic acid were ranged from 19.47 µg/g in the sample collected from Huairou, Beijing to 62.61 µg/g in the sample collected from Jixian, Tianjin. Overall, chestnut samples from Central China contained relatively higher mean value of gallic acid than the samples from other regions. However, chestnut samples collected from South China contained the minimum mean value of gallic acid.

With regard to gentisic acid, levels ranged from 8.95 µg/g in samples collected from Tangshan, Hebei to 35.15 µg/g in samples collected from Fuzhou, Jiangxi. The highest mean value of gentisic acid was found in East China, and samples collected from Fuzhou, Jiangxi were shown as much higher value of gentisic acid (35.15 µg/g) than other cities in the group of East China. The similar tendency was also observed in vanillic acid and caffeic acid. In terms of 2,3,4-trihydroxybenzoic acid, contents were ranged from 5.71 µg/g in samples collected from Wenzhou, Zhejiang to 9.45 µg/g in samples collected from Haikou, Hainan. No results were detected in samples collected from several cities, including Tangshan and Qinhuangdao (North China), Linyi (East China), Xinyang (Central China), Liuzhou (South China), and Kunming (Southwest China).

Contents of protocatechuic acid were ranged from 5.97 µg/g in the sample collected from Xinyang, Henan to 51.78 µg/g in the sample collected from Fuzhou, Jiangxi. Overall, samples collected from East China contained highest average value of protocatechuic acid while that from Central China contained the lowest value. The similar tendency was also observed in *p*-hydroxybenzoic acid. The content of *p*-hydroxybenzoic acid were ranged from 2 µg/g in the samples collected from Jixian, Tianjin to 27.01 µg/g in the samples collected from Fuzhou, Jiangxi. For chlorogenic acid, contents were ranged from 3.40 µg/g in samples collected from Kunming, Yunnan to 22.86 µg/g in the samples collected from Anqing, Anhui.

In terms of protocatechualdehyde, syringic acid, *p*-coumaric acid and syringaldehyde, ferulic acid, and sinapic acid, all detected contents were pretty low (typically below 10 µg/g). The highest level of protocatechualdehyde and ferulic acids were found in Southern regions. However, more synaptic acid and *p*-coumaric acid were observed in Central China. The content of syringic acid ranged from 2.43 µg/g to 10.05 µg/g, and was observed most in East China. Overall, contents of different phenolic compounds were significantly different (*p* < 0.05) from different regions.

## 4. Discussion

### 4.1. Phenolic and Antioxidant Properties among 21 Raw Chestnut Samples

This study has revealed that all the chestnut samples under investigation are rich sources of TPC, TFC, and CTC. These samples have also presented high antioxidant activities as assessed by DPPH, FRAP, and ABTS assay. It has been well reported in the literature that phenolic compounds prevent cardiovascular diseases, cataract development, oxidative injury caused by heat stress, lower the incidence of influenza infection, reduce fat absorption, and enhance energy expenditure [9,19]. Overall, significant differences (*p* < 0.05) were observed among the phenolic profiles and antioxidant activities of all chestnut samples procured from five different geographic regions of China. As mentioned in Table 3, raw chestnut samples from in South China presented relatively higher values of TPC, CTC, and antioxidant activities in terms of DPPH, FRAP, and ABTS values compared to samples collected from other regions. However, the samples from Southwest China presented relatively higher TFC values but contain lower level of TPC, DPPH, FRAP, and ABTS values compared to the samples from other geographical regions.

In particular, samples from Fuzhou (East China) exhibit higher values corresponding to TPC (2.35 mg GAE/g), DPPH (16.74 μmol TE/g), FRAP (3.20 mmol FE/100 g), and ABTS (24.83 μmol TE/g) assays. Previously, a study has reported 2.84 mg GAE/g of TPC in chestnut fruits collected from Tenerife, Spain (C. sativa Mill) [20] Whereas, Otles and Selek [21] mentioned even higher TPC values from 5.00 to 32.82 mg GAE/g in Turkish chestnuts (*C. sativa* Mill). The relatively lower phenolic contents of chestnuts investigated in this study may be attributed to the certain degree of oxidation of raw chestnuts peeled by chestnut peeler. Earlier, chestnuts from Italy reported to exhibit lower values for ABTS (4.77 to 8.15 μmol TE/g) compared to the present study [22]. The samples from Kunming (Southwest China) presented the highest values for TFC (1.13 mg CAE/g). However, earlier 2.62 mg CAE/g of TFC was reported in Spanish chestnuts (*C. sativa* Mill) [6].

Furthermore, in spite of differences among the phenolic profile of samples from different geographic regions, inter-provincial and intra-provincial disparities among the TPC, TFC, CTC, and antioxidant values of all the chestnut samples were also observed. The samples collected from Qinhuangdao, Hebei presented relatively higher values for phenolics and antioxidant capacities compared to the samples procured from Tangshan and Xingtai cities of Hebei Province. However, samples from Tangshan and Xingtai of Hebei Province presented less difference among the TPC, TFC, CTC, DPPH and ABTS values except for the FRAP value (2.13 mmol FE/100 g in case of samples from Tangshan and 1.40 mmol FE/100 g in case of samples from Xingtai). A similar phenomenon was also presented by the samples collected from South China. The samples from Guilin and Liuzhou of Guangxi Province exhibit comparatively less difference in TPC, TFC, DPPH, and FRAP values. However, as shown in Table 3, a considerable difference was observed in CTC and ABTS values of samples collected from these two different regions of Guangxi Province. Nevertheless, the samples from Zhaotong and Kunming of Yunnan Province exhibit significant differences among the values of all the assays. The TFC (1.13 mg CAE/g) and CTC (10.69 mg CAE/g) values of samples procured from Kunming were about two times compared to TFC (0.63 mg CAE/g) and CTC (4.57 mg CAE/g) values presented by samples from Zhaotong. However, the ABTS value of samples from Kumming was observed to be approximately three times higher than the ABTS value presented by samples from Zhaotong. The samples procured from Taian and Linyi region of Shandong Province also revealed significant differences among all the values related to phenolics and antioxidant assays. It was also observed that the samples from Taian exhibit significantly higher ABTS values (16.71 μmol TE/g) compared to samples collected from Linyi region (12.55 μmol TE/g).

Based on these findings, it may be concluded that differences among the phenolic profiles and antioxidant activities of chestnut samples significantly depend on the geographical factors, such as temperature and sunlight exposure. The correlation between average temperature and sunlight with phenolic contents and antioxidant capacities were shown in Supplemental Figure S4. Higher average temperature during growing period (27.26 °C in South China) of chestnut helps present higher phenolic contents (in terms of TPC and CTC) and antioxidant activities. This may due to the elevated temperature could facilitate photosynthetic capacity of plants to produce more secondary metabolites, such as phenolic acids [23]. Chestnuts grown in Fuzhou, Jiangxi, although belong to East China (average temperature = 23.72 °C), its geographical location is very near the South China and the average temperature reaches to 26.33 ˚C during growing period [16]. On the other side, more sunlight duration (213.12 h/month in North China) also helps improve antioxidant activities in terms of DPPH and FRAP. i.e., Chestnut samples from North China (213.12 h/month) exhibited higher average values than Central China (180.84 h/month) and Southwest China (162.02 h/month). In response to high levels of sunlight, plants are able to adapt to the circumstances and release various secondary metabolites including phenolic compounds and triterpenoids, which have well-known antioxidant properties [24,25]. However, TFC value is less affected by these geographical factors. This finding is in agreement with results of the previous study that reported a significant difference in flavonoids and phenolic content of Chinese chestnut collected from various ecological regions [15]. Compared with other chestnut varieties, *Castanea sativa* Mill (European variety) were found to present the highest performance in net photosynthesis with higher temperature (26 °C) in September [23]. This was also in agreement with results from Almeida et al. [26], in which the optimal temperature for the highest rates of net photosynthesis of chestnut (*C. sativa* Mill) were in a range of 31 to 33.5 °C.

### 4.2. Analysis of Phenolic and Antioxidant Contents Based on Morphological Features

The mean values of TPC, TFC, CTC, and antioxidant activities of three identified sub-groups of chestnut, *Banli*, *Maoli*, and *Youli* are described in Table 6. Overall, the phenolic profile and antioxidant activities exhibit significant differences (*p* < 0.05) among these three sub-groups. It was also observed that *Banli* presented the highest values for TPC, TFC, CTC, and antioxidant capacities as assessed by DPPH, FRAP, and ABTS assays, followed by *Maoli* and *Youli*.

Specifically, among all *Banli* varieties, chestnuts from Guilin and Liuzhou of Guangxi Province (South China) contributed considerably towards the higher level of phenolics and antioxidant activities. The *Banli* samples collected from Kunming (Southwest China) observed to impose a major impact on the overall high TFC level of *Banli* variety. The *Banli* samples from Xiangyang, Hubei (Central China) presented the highest ABTS value (19.86 μmol TE/g) compared to other samples.

Amongst *Maoli* varieties, samples from Fuzhou, Jiangxi (East China) exhibit a major contribution towards the high mean values of all the assays employed to determine the phenolic profile and antioxidant capacities. On the other hand, chestnuts from Zhaotong, Yunnan (Southwest China) exhibited the lowest values in TPC, TFC, CTC, DPPH, and ABTS assays.

In case of *Youli* varieties, samples from Wenzhou, Zhejiang (East China) presented relatively higher values for TPC, TFC, CTC, DPPH, and ABTS assays. However, *Youli* samples from Xinyang, Henan (Central China) contain relatively lower values for TPC, TFC, CTC and antioxidant activities determined by DPPH, FRAP, and ABTS assay. These results are also in agreement with conclusion that phenolic profiles and antioxidant activities are largely depend on other geographical factors not limited to sample varieties.

### 4.3. Correlation among Phenolic Contents, Antioxidant Activities, and Color Values

The correlation coefficient (r) between phenolic compounds, antioxidant capacities, and color values has also been established and presented in Table 7. The strong and positive correlations were observed in phenolic profiles of chestnut samples in terms of TPC, TFC, and CTC. The highest correlation coefficient value was found between TPC and CTC (r = 0.834, *p* < 0.01), followed by between TPC and TFC (r = 0.762, *p* < 0.01) and between TFC and CTC (r = 0.708, *p* < 0.01). Additionally, the stronger positive correlation was also exhibited between three antioxidant assays. The highest correlation coefficient (r) was determined as 0.875 (*p* < 0.01) between DPPH and ABTS values, followed by 0.819 (*p* < 0.01) between FRAP and DPPH as well as between FRAP and ABTS values. All the parameters related to phenolic contents and antioxidant capacities have presented a positive linear correlation with each other. The highest correlation value was shown as 0.884 (*p* < 0.01) between TPC and ABTS. A comparatively low and positive correlation was found between TFC and FRAP (r = 0.540, *p* < 0.05). For color values, no significant correlations were found between lightness (*L*) and other phenolic and antioxidant parameters.

### 4.4. Analysis of Phenolic Acid Profile Based on Geographic Regions

Overall, chestnut samples from all five regions of China were abundant with phenolic acids. The beneficial function of phenolic acid has been illustrated in this article, including preventing cancer, heart disease, and cardiovascular disease [27]. In all 14 phenolic acids detected in this study, gallic acid and vanillin were two most predominant phenolic acids found in chestnuts, which in accordance with the results from the research conducted by Otles and Selek [21]. However, three phenolic acids, 2,3,4-trihydroxybenzoic acid, protocatechualdehyde and sinapic acid, were found the least values in chestnut samples collected in China.

Based on different geographic areas of China, chestnut samples collected from East China contained the highest total phenolic acids (211.37 µg/g), followed by South China (188.72 µg/g) and North China (183.85 µg/g). Central China contained the fewest overall phenolic acids (168.81 µg/g). Phenolic acids in samples collected from different regions varied significantly, mainly attributed to both geographical factors and some human factors. From the perspective of geographical factors, adequate exposure to sunlight and moderate precipitation contributed to higher value of phenolic acid inside plants [25]. Especially, Fuzhou, Jiangxi (East China) exhibited overwhelmingly high contents of vanillin (70.72 µg/g), gallic acid (55.08 µg/g) and protocatechuic acid (51.78 µg/g). The higher contents of chestnuts collected from Fuzhou may attributed to warmer temperature (26.33 °C) and adequate precipitation (1600 mm) during growing seasons. The results observed by HPLC were also in accordance with the previous colorimetric assays.

Taking a deeper look at the different types of phenolic acids, vanillin was the most abundant one among other phenolic acids observed in chestnuts samples collected from China. The higher contents of vanillin were found in South China (61.05 µg/g) and North China (61.47 µg/g), due to the warmer average temperature (27.26 °C in South China) and sufficient sunlight exposure (213.12 h/month in North China). It is delightful to observe higher amount of vanillin in chestnut samples as vanillin has been proved to possess potent antioxidant capacity [28]. According to Clemens et al. [29], vanillin is also shown to have some beneficial health effects to human, such as inhibiting lipid oxidation, preventing DNA damage from exposure to excessive sunlight, preventing the forming of cancer, etc. Besides, Sanz et al. [30] has proved that toasting will lead to the degradation of lignin, and promote releasing of low-molecular weight phenolic compound, such as vanillin. Therefore, higher contents of vanillin found in chestnut samples (99.33 µg/g) collected from Haikou, Hainan could be explained by sufficient sunlight exposure and higher average temperature during the growing season.

In terms of gallic acid, it is known as having anti-inflammatory, anti-microbial and radical scavenging activities which can be very helpful in treating diseases including cancer, asthma, Alzheimer, and so on according to [31]. In contrast to vanillin, the highest contents of gallic acid were found in Central China (42.97 µg/g) and East China (41.69 µg/g). A reasonable explanation for this can also be found in research conducted by Sanz et al. [30], which illustrated that gallic acid was very sensitive to heat, thus decomposition of gallic acid may occur with higher temperature.

### 4.5. Analysis of Phenolic Acid Profile Based on Morphological Features

Mean values of 14 phenolic acids of three types of chestnuts based on morphological features were described in Table 8. Overall, the total phenolic acid content among three chestnut varieties were significantly differed (*p* < 0.05), and *Youli* presented the highest value (206.53 µg/g), followed by *Banli* (193.43 µg/g) and *Maoli* (176.78 µg/g).

Specifically, in terms of *Youli* varieties, the extremely high value of total phenolic content (314 µg/g) was observed in chestnut samples collected from Fuzhou, Jiangxi, which becomes the key factor for higher value of this variety. However, there is only two out of five of *Youli* variety chestnut samples exceeded the average total phenolic acid value (206.53 µg/g). The lowest value of phenolic acid among 21 chestnuts was observed in samples collected from Xinyang, Henan (142.35 µg/g).

With regard to *Banli* varieties, five out of ten chestnut samples exceeded the mean value (193.43 µg/g). The major contributors to higher phenolic content of *Banli* variety are samples collected from Haikou, Hainan (240.43 µg/g) and Anqing, Anhui (237.67 µg/g). The lowest total phenolic acid content among *Banli* varieties was observed in Kunming, Yunnan (154.11 µg/g).

In terms of *Maoli* varieties, the mean phenolic acid content was the lowest among three varieties which is mainly due to the sample collected from Taian, Shandong (144.28 µg/g). Based on the above-mentioned findings, it could be seen that no observable relationships between phenolic acid content and morphological features were found.

## 5. Conclusions

The phenolic profile and antioxidant activity of chestnut from five different geographical areas of China have been explored in this study. All the samples from different regions and varieties exhibit significant difference (*p* < 0.05) in TPC, TFC, CTC, DPPH, FRAP, and ABTS values. It was observed that the chestnut samples from Fuzhou, Jiangxi (East China) exhibited the higher level of TPC (2.35 mg GAE/g) and CTC (13.52 mg CAE/g) and antioxidant activity among all the chestnut samples, and also exhibited the highest total phenolic acid content (314.87 µg/g). However, the samples collected from Kunming, Yunnan (Southwest China) presented the highest level of TFC (1.13 mg CAE/g). Among the five geographical regions, samples from South China revealed maximum mean values for TPC (1.89 mg GAE/g), CTC (9.41 mg CAE/g), DPPH (11.76 μmol TE/g), and FRAP (2.37 mmol FE/100 g). Whereas the samples from Southwest China exhibit minimum mean values for TPC (1.41 mg GAE/g), DPPH (8.30 μmol TE/g), FRAP (0.99 mmol FE/100 g) and ABTS (11.52 μmol TE/g). Among 14 free phenolic compounds, vanillin and gallic acid were found to be most abundant. The content of vanillin is more in warmer regions because high temperature may lead to decomposition of lignin and release more phenolic compounds. Higher temperature and more sunlight exposure during growing period of chestnuts help to improve phenolic profiles and antioxidant activities of chestnut samples. Among three varieties of chestnut, *Banli* presented higher mean values for TPC, TFC, CTC, and antioxidant capacities, followed by *Maoli* and *Youli*. However, no observable relationships between phenolic acid content and morphological features were found. Overall, chestnuts samples exhibit a considerable number of phenolic compounds and potent antioxidant activities. The significant variations in phenolic compounds and antioxidant activity were observed based on the geographical regions and varieties of chestnuts. The findings of this study will have a major importance for the consumers, food scientists, plant breeders and commercial chestnut growers for the better selection of specific chestnut variety from a particular geographical region for maximum health benefits, production of functional food, developing high-value chestnut varieties and selection of geographical site for further cultivation of chestnut plants. In future study, the effect on the thermal processing of chestnuts from different geographical areas will be further investigated.

## Figures and Tables

**Table 1:** Information of chestnut samples in different parts of China.

Code	Variety	Common Name	Size	Growing Area	Region	Average Temperature (°C)	Average Sunlight Duration (h/month)
1	*Castanea mollissima*	*Banli*	Medium	Huairou, Beijing	North China	22.33	213.12
2	*Castanea mollissima*	*Youli*	Small	Jixian, Tianjin			
3	*Castanea mollissima*	*Banli*	Medium	Tangshan, Hebei			
4	*Castanea mollissima*	*Banli*	Medium	Xingtai, Hebei			
5	*Castanea mollissima*	*Banli*	Medium	Qinhuangdao, Hebei			
6	*Castanea mollissima*	*Maoli*	Small	Shangluo, Shanxi	Northwest China		
7	*Castanea mollissima*	*Banli*	Medium	Wuxi, Jiangsu	East China	23.72	182.82
8	*Castanea mollissima*	*Youli*	Medium	Wenzhou, Zhejiang			
9	*Castanea mollissima*	*Banli*	Medium	Anqing, Anhui			
10	*Castanea mollissima*	*Youli*	Medium	Nanping, Fujian			
11	*Castanea mollissima*	*Maoli*	Small	Fuzhou, Jiangxi			
12	*Castanea mollissima*	*Maoli*	Small	Taian, Shandong			
13	*Castanea mollissima*	*Maoli*	Small	Linyi, Shandong			
14	*Castanea mollissima*	*Youli*	Medium	Xinyang, Henan	Central China	23.82	180.84
15	*Castanea mollissima*	*Banli*	Medium	Xiangyang, Hubei			
16	*Castanea mollissima*	*Youli*	Large	Yangjiang, Guangdong	South China	27.26	185.05
17	*Castanea mollissima*	*Banli*	Medium	Guilin, Guangxi			
18	*Castanea mollissima*	*Banli*	Medium	Liuzhou, Guangxi			
19	*Castanea mollissima*	*Maoli*	Small	Haikou, Hainan			
20	*Castanea mollissima*	*Maoli*	Small	Zhaotong, Yunnan	Southwest China	21.21	162.02
21	*Castanea mollissima*	*Banli*	Medium	Kunming, Yunnan			

**Table 2:** Yield, moisture content, and color value of chestnuts from different geographic areas.

Code	Region	Growing Area	Yield	Moisture Content (%)	Color Value		
					*L*	a*	b*
1	North China	Huairou, Beijing	61.17%	9.15 ± 0.00 ^d,e^	88.81 ^g^	−1.53 ^q^	11.39 ^d,e^
2		Jixian, Tianjin	39.11%	13.14 ± 0.00 ^a^	91.04 ^e,f^	−0.83 ^k^	10.94 ^g,h^
3		Tangshan, Hebei	46.92%	10.21 ± 0.00 ^c^	91.04 ^e,f^	−0.36 ^d^	9.03 ^o^
4		Xingtai, Hebei	58.38%	9.17 ± 0.01 ^d,e^	90.56 ^e,f^	−0.28 ^c^	11.72 ^c^
5		Qinhuangdao, Hebei	56.55%	7.24 ± 0.00 ^h^	92.57 ^a,b,c^	−0.95 ^m^	10.76 ^h,i^
6	Northwest China	Shangluo, Shanxi	42.23%	7.54 ± 0.00 ^g,h^	90.77 ^e,f^	−0.89 ^l^	11.09 ^f,g^
7	East China	Wuxi, Jiangsu	44.86%	5.76 ± 0.00 ^i,j^	91.62 ^c,d,e^	−1.00 ^n^	9.76 ^n^
8		Wenzhou, Zhejiang	49.55%	9.56 ± 0.01 ^c,d^	93.51 ^a^	−0.06 ^b^	7.63 ^q^
9		Anqing, Anhui	48.68%	5.02 ± 0.00 ^j^	93.39 ^a^	−0.44 ^e^	8.57 ^p^
10		Nanping, Fujian	51.90%	8.95 ± 0.01 ^d,e,f^	90.98 ^e,f^	−0.70 ^i^	11.14 ^e,f,g^
11		Fuzhou, Jiangxi	54.70%	8.37 ± 0.01 ^e,f,g^	85.77 ^h^	0.50 ^a^	10.16 ^m^
12		Taian, Shandong	47.16%	10.30 ± 0.01 ^c^	91.55 ^c,d,e^	−0.42 ^e^	10.70 ^h,i,j^
13		Linyi, Shandong	52.06%	8.24 ± 0.00 ^e,f,g^	92.17 ^b,c,d^	−1.25 ^p^	10.28 ^l,m^
14	Central China	Xinyang, Henan	49.97%	11.54 ± 0.01 ^b^	92.57 ^a,b,c^	−1.81 ^r^	13.00 ^b^
15		Xiangyang, Hubei	53.16%	6.21 ± 0.01 ^i^	90.81 ^e,f^	−1.04 ^o^	11.46 ^d^
16	South China	Yangjiang, Guangdong	47.03%	5.87 ± 0.00 ^i,j^	92.95 ^a,b^	−2.52 ^s^	14.94 ^a^
17		Guilin, Guangxi	51.27%	7.66 ± 0.00 ^g,h^	90.80 ^e,f^	−0.74 ^j^	10.59 ^i,j,k^
18		Liuzhou, Guangxi	47.19%	8.16 ± 0.00 ^f,g,h^	90.00 ^f^	−0.58 ^g^	11.41 ^d,e^
19		Haikou, Hainan	49.78%	7.43 ± 0.01 ^g,h^	91.40 ^d,e^	−0.65 ^h^	10.46 ^j,k,l^
20	Southwest China	Zhaotong, Yunnan	54.65%	8.69 ± 0.00 ^d,e,f^	90.10 ^f^	−0.91 ^l^	11.35 ^d,e,f^
21		Kunming, Yunnan	50.76%	8.99 ± 0.01 ^d,e,f^	90.22 ^f^	−0.561 ^f^	10.35 ^k,l,m^

Values are expressed as the mean of triplicates ± standard deviation. Means in the same column with unlike superscripts (^a–s^) differ significantly. (*p* < 0.05).

**Table 3:** Phenolic profiles (total phenolic contents (TPC), total flavonoids content (TFC), and condensed tannin content (CTC)), and antioxidant capacities (DPPH free radical scavenging capacity (DPPH), ferric reducing antioxidant power (FRAP), and ABTS free radical scavenging capacities (ABTS)) of chestnut samples.

Code	Region	Growing Area	TPC (mg GAE/g)	TFC (mg CAE/g)	CTC (mg CAE/g)	DPPH (μmol TE/g)	FRAP (mmol FE/100 g)	ABTS (μmol TE/g)
1	North China	Huairou, Beijing	1.18 ± 0.11 ^k,l^	0.60 ± 0.03 ^j,k^	4.99 ± 0.49 ^i^	8.60 ± 0.26 ^g,h^	0.82 ± 0.06 ^g^	9.45 ± 0.84 ^h^
2		Jixian, Tianjin	1.53 ± 0.14 ^h,i^	0.65 ± 0.06 ^h,i,j,k^	7.79 ± 0.41 ^f^	9.86 ± 0.48 ^d,e,f^	1.97 ± 0.04 ^d^	12.36 ± 0.41 ^e,f^
3		Tangshan, Hebei	1.59 ± 0.09 ^f,g,h,i^	0.78 ± 0.03 ^e,f,g^	6.81 ± 0.20 ^g,h^	10.18 ± 0.39 ^d,e^	2.13 ± 0.18 ^d^	14.31 ± 0.46 ^d^
4		Xingtai, Hebei	1.58 ± 0.13 ^f,g,h,i^	0.68 ± 0.01 ^g,h,i,j,k^	7.42 ± 0.42 ^f,g^	10.02 ± 0.10 ^d,e^	1.40 ± 0.12 ^e^	13.08 ± 0.43 ^d,e^
5		Qinhuangdao, Hebei	1.92 ± 0.15 ^c,d,e^	0.84 ± 0.05 ^d,e^	11.15 ± 0.83 ^c^	12.81 ± 0.06 ^c^	3.10 ± 0.29 ^a^	17.69 ± 1.49 ^c^
21	Northwest China	Shangluo, Shanxi	1.50 ± 0.09 ^h,i,j^	0.75 ± 0.07 ^e,f,g,h^	5.27 ± 0.41 ^i^	10.00 ± 0.20 ^d,e^	1.48 ± 0.04 ^e^	11.72 ± 0.60 ^e,f,g^
	Mean ± SD		1.55 ± 0.24	0.72 ± 0.09	7.24 ± 2.22	10.25 ± 1.38	1.82 ± 0.78	13.10 ± 2.77
6	East China	Wuxi, Jiangsu	1.38 ± 0.11 ^i j k^	0.68 ± 0.05 ^g,h,i,j^	6.33 ± 0.49 ^h^	9.12 ± 0.43 ^f,g^	0.73 ± 0.05 ^g^	10.44 ± 0.66 ^g,h^
7		Wenzhou, Zhejiang	1.80 ± 0.18 ^d,e,f^	0.97 ± 0.06 ^b,c^	9.97 ± 0.68d ^e^	10.12 ± 0.48 ^d,e^	1.55 ± 0.04 ^e^	12.53 ± 1.20 ^e,f^
8		Anqing, Anhui	1.97 ± 0.11 ^b,c,d^	0.91 ± 0.08 ^c,d^	12.12 ± 0.66 ^b^	10.16 ± 0.24 ^d,e^	2.68 ± 0.15 ^b^	16.82 ± 0.87 ^c^
9		Nanping, Fujian	1.29 ± 0.08 ^j,k^	0.57 ± 0.03 ^k^	3.43 ± 0.34 ^j^	7.97 ± 0.58 ^h,i^	1.07 ± 0.02 ^f^	10.67 ± 0.51 ^g,h^
10		Fuzhou, Jiangxi	2.35 ± 0.22 ^a^	1.01 ± 0.08 ^b^	13.52 ± 1.10 ^a^	16.74 ± 0.92 ^a^	3.20 ± 0.26 ^a^	24.83 ± 0.19 ^a^
11		Taian, Shandong	1.68 ± 0.13 ^e,f,g,h^	0.82 ± 0.06 ^d,e,f^	6.76 ± 0.54 ^g,h^	10.42 ± 0.35 ^d^	2.05 ± 0.15 ^d^	16.71 ± 1.63 ^c^
12		Linyi, Shandong	1.40 ± 0.01 ^i,j,k^	0.65 ± 0.08 ^h,i,j,k^	9.43 ± 0.91 ^e^	9.38 ± 0.13 ^e,f^	0.66 ± 0.03 ^g^	12.55 ± 0.18 ^e,f^
	Mean ± SD		1.70 ± 0.38	0.80 ± 0.17	8.79 ± 3.52	10.56 ± 2.85	1.71 ± 0.98	14.94 ± 5.07
13	Central China	Xinyang, Henan	1.19 ± 0.06 ^k,l^	0.63 ± 0.05 ^j,k^	4.26 ± 0.33 ^i,j^	7.49 ± 0.27 ^i,j^	1.38 ± 0.09 ^e^	9.51 ± 0.72 ^h^
14		Xiangyang, Hubei	1.92 ± 0.19 ^c,d,e^	0.85 ± 0.08 ^d,e^	12.07 ± 0.23 ^b^	10.45 ± 0.66 ^d^	2.38 ± 0.08 ^c^	19.86 ± 0.85 ^b^
	Mean ± SD		1.56 ± 0.52	0.74 ± 0.16	8.17 ± 5.52	8.97 ± 2.09	1.88 ± 0.71	14.69 ± 7.32
15	South China	Yangjiang, Guangdong	1.54 ± 0.14 ^g,h,i^	0.70 ± 0.04 ^g,h,i,j^	5.26 ± 0.48 ^i^	10.10 ± 0.57 ^d,e^	1.36 ± 0.12 ^e^	11.26 ± 0.69 ^f,g^
16		Guilin, Guangxi	2.19 ± 0.10 ^a,b^	1.03 ± 0.03 ^b^	12.33 ± 0.33 ^b^	13.87 ± 0.70 ^b^	3.24 ± 0.12 ^a^	17. 92 ± 0.59 ^c^
17		Liuzhou, Guangxi	2.12 ± 0.19 ^a,b,c^	0.71 ± 0.04 ^g,h,i,j^	13.58 ± 0.57 ^a^	13.62 ± 0.27 ^b^	2.77 ± 0.13 ^b^	19.61 ± 1.01 ^b^
18		Haikou, Hainan	1.72 ± 0.17 ^d,e,f,g,h^	0.74 ± 0.04 ^f,g,h,i^	6.48 ± 0.31 ^g,h^	9.44 ± 0.33 ^e,f^	2.12 ± 0.11 ^d^	13.16 ± 1.32 ^d,e^
	Mean ± SD		1.89 ± 0.31	0.80 ± 0.16	9.41 ± 4.15	11.76 ± 2.31	2.37 ± 0.82	14.68 ± 8.16
19	Southwest China	Zhaotong, Yunnan	1.03 ± 0.05 ^l^	0.63 ± 0.05 ^i,j,k^	4.57 ± 0.27 ^i^	7.08 ± 0.15 ^j^	0.84 ± 0.06 ^g^	6.53 ± 0.35 ^i^
20		Kunming, Yunnan	1.79 ± 0.14 ^d,e,f,g^	1.13 ± 0.07 ^a^	10.69 ± 0.59 ^c,d^	9.52 ± 0.14 ^e,f^	1.13 ± 0.11 ^f^	16.51 ± 1.23 ^c^
	Mean ± SD		1.41 ± 0.54	0.88 ± 0.35	7.63 ± 4.33	8.30 ± 1.73	0.99 ± 0.21	11.52 ± 7.06

Values are expressed as the mean of triplicates ± standard deviation. Means in the same column with unlike superscripts (^a–l^) differ significantly (*p* < 0.05).

**Table 4:** Chemical structures of phenolic compounds.

Name	Structure	Name	Structure
Gallic acid	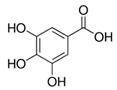	Vanillic acid	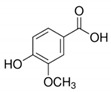
Protocatechuic acid	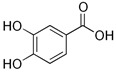	Caffeic acid	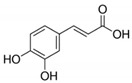
2,3,4-Trihydroxybenzoic acid	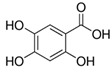	Syringic acid	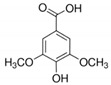
Protocatechualdehyde	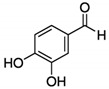	Vanillin	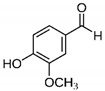
*p*-Hydroxybenzoic acid	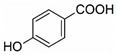	*p*-Coumaric acid	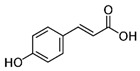
Gentisic acid	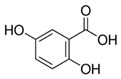	Ferulic acid	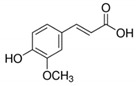
Chlorogenic acid	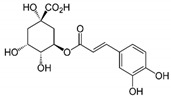	Sinapic acid	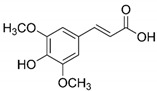

**Table 5:** Phenolic acids content of 21 chestnut samples.

Region	Gallic Acid	Protocatechuic Acid	2,3,4-Trihydroxybenzoic Acid	Protocatechualdehyde	*p*-Hydroxybenzoic Acid	Gentisic Acid	Chlorogenic Acid	Vanillic Acid + Caffeic Acid	
Huairou, Beijing	19.47 ± 0.61 ^m^	11.87 ± 0.47 ^h^	7.46 ± 0.28 ^c^	1.85 ± 0.14 ^d^	7.05 ± 0.46 ^h,i^	11.77 ± 0.53 ^k^	13.07 ± 0.16 ^c^	22.64 ± 0.40 ^h,i^	North China
Jixian, Tianjin	62.61 ± 0.38 ^a^	21.82 ± 0.25 ^c^	6.47 ± 0.02 ^e,f^	1.44 ± 0.07 ^g^	2.00 ± 0.08 ^l^	9.16 ± 0.32 ^m,n^	10.50 ± 0.14 ^d,e^	5.04 ± 0.07 ^p^	
Tangshan, Hebei	28.96 ± 1.44 ^k,l^	11.97 ± 0.62 ^g,h^	ND	1.38 ± 0.03 ^g,h^	7.71 ± 0.17 ^g,h^	8.95 ± 0.57 ^n^	4.14 ± 0.13 ^n^	28.93 ± 0.45 ^f^	
Xingtai, Hebei	40.44 ± 2.67 ^e^	12.70 ± 1.01 ^g,h^	6.13 ± 0.02 ^h^	1.63 ± 0.01 ^f^	7.92 ± 0.10 ^g,h^	16.90 ± 1.59 ^h,i^	8.57 ± 0.30 ^g^	30.12 ± 0.96 ^e^	
Qinhuangdao, Hebei	34.43 ± 0.61 ^h^	19.02 ± 0.06 ^d^	ND	1.19 ^j,k^	11.62 ± 0.32 ^d,e^	9.52 ± 0.33 ^m,n^	4.42 ± 0.01 ^n^	20.57 ± 0.47 ^j^	
Shangluo, Shanxi	26.97 ± 1.04 ^l^	12.37 ± 0.26 ^g,h^	6.41 ± 0.01 ^e,f,g^	2.10 ± 0.04 ^b^	6.43 ± 0.24 ^i,j^	15.34 ± 0.71 ^j^	7.85 ± 0.28 ^h,i^	24.46 ± 0.13 ^g^	Northwest China
Mean ± SD	35.48 ± 1.13	14.96 ± 0.45	4.41 ± 0.08	1.60 ± 0.06	7.12 ± 0.23	11.94 ± 0.68	8.09 ± 0.17	21.96 ± 0.41	
Wuxi, Jiangsu	35.56 ± 0.63 ^f^	17.19 ± 1.32 ^e,f^	8.00 ± 0.25 ^b^	1.79 ± 0.05 ^d,e^	5.90 ± 0.54 ^j^	20.05 ± 0.61 ^d,e^	7.33 ± 0.36 ^i,j^	23.68 ± 0.49 ^g,h^	East China
Wenzhou, Zhejiang	46.00 ± 0.84 ^d^	19.31 ± 0.62 ^d^	5.71 ^k^	2.30 ± 0.01 ^a^	8.14 ± 0.54 ^g^	18.96 ± 0.43 ^e,f^	6.31 ± 0.11l ^m^	35.48 ± 0.86 ^b,c^	
Anqing, Anhui	40.39 ± 0.21 ^e^	26.48 ± 0.70 ^b^	6.32 ± 0.02 ^g^	1.74 ± 0.02 ^e^	12.70 ± 0.58 ^b,c^	21.50 ± 0.81 ^c^	22.86 ± 0.10 ^a^	37.98 ± 0.53 ^a^	
Nanping, Fujian	39.64 ± 1.26 ^e,f^	8.94 ± 0.30 ^i^	6.35 ± 0.04 ^f,g^	1.95 ± 0.07 ^c^	10.31 ± 0.40 ^f^	11.05 ± 0.23 ^k,l^	6.79 ± 0.18 ^k,l^	30.68 ± 1.36 ^e^	
Fuzhou, Jiangxi	55.08 ± 0.57 ^b^	51.78 ± 2.19 ^a^	5.83 ± 0.05 ^j,k^	2.21 ± 0.10 ^a^	27.01 ± 1.31 ^a^	35.15 ± 0.13 ^a^	10.23 ± 0.83 ^e^	36.57 ± 1.20 ^b^	
Taian, Shandong	39.98 ± 1.94 ^e^	7.17 ± 0.25 ^j,k^	5.97 ± 0.03 ^i,j^	0.60 ± 0.03 ^m^	5.57 ± 0.34 ^j^	9.72 ± 0.51 ^m,n^	6.17 ± 0.27 ^m^	22.81 ± 1.01 ^h,i^	
Linyi, Shandong	35.18 ± 0.41 ^h^	16.15 ± 0.35 ^f^	ND	1.97 ± 0.01 ^c^	8.64 ± 0.17 ^g^	18.63 ± 0.56 ^f,g^	7.37 ± 0.36 ^i,j^	33.10 ± 0.42 ^d^	
Mean ± SD	41.69 ± 0.84	21.00 ± 0.82	5.45 ± 0.07	1.80 ± 0.04	11.18 ± 0.55	19.30 ± 0.47	9.58 ± 0.32	31.47 ± 0.84	
Xinyang, Henan	48.42 ± 3.14 ^c^	5.97 ± 0.14 ^k^	ND	1.12 ± 0.04 ^k^	ND	17.58 ± 0.80 ^g,h^	7.05 ± 0.22 ^j,k^	17.14 ± 0.60 ^m^	Central China
Xiangyang, Hubei	37.51 ± 1.55 ^f^	13.40 ± 0.45 ^g^	6.55 ± 0.05 ^e^	1.25 ± 0.05 ^i,j^	11.09 ± 0.56 ^e,f^	11.45 ± 1.14 ^k^	6.33 ± 0.31 ^l,m^	18.49 ± 0.60 ^l^	
Mean ± SD	42.97 ± 2.34	9.68 ± 0.30	3.28 ± 0.05	1.19 ± 0.05	5.55 ± 0.56	14.52 ± 0.97	6.69 ± 0.26	17.82 ± 0.60	
Yangjiang, Guangdong	39.12 ± 0.93 ^e,f^	8.35 ± 0.12 ^i,j^	6.10 ± 0.05 ^h,i^	1.56 ± 0.06 ^f^	3.62 ± 0.18 ^k^	27.61 ± 0.89 ^b^	7.31 ± 0.28 ^i,j,k^	22.24 ± 0.62 ^i^	South China
Guilin, Guangxi	31.97 ± 0.37 ^i,j^	16.95 ± 1.66 ^e,f^	7.26 ± 0.13 ^d^	0.91 ± 0.01 ^l^	13.57 ± 1.31 ^b^	11.78 ± 0.26 ^k^	10.81 ± 0.07 ^d^	15.88 ± 0.47 ^n^	
Liuzhou, Guangxi	29.19 ± 2.15 ^k,l^	18.01 ± 1.73 ^d,e^	ND	1.31 ± 0.09 ^h,i^	7.94 ± 0.78 ^g,h^	18.34 ± 0.85 ^f,g^	8.30 ± 0.53 ^g,h^	13.98 ± 0.91 ^o^	
Haikou, Hainan	30.24 ± 0.60 ^j,k^	16.87 ± 0.45 ^e,f^	9.45 ± 0.08 ^a^	2.31 ± 0.03 ^a^	12.17 ± 0.34 ^c,d^	10.12 ± 0.55 ^l,m^	16.64 ± 0.50 ^b^	18.92 ± 0.38 ^k,l^	
Mean ± SD	32.63 ± 1.01	15.05 ± 0.99	5.70 ± 0.09	1.52 ± 0.05	9.33 ± 0.65	16.96 ± 0.64	10.77 ± 0.34	17.76 ± 0.59	
Zhaotong, Yunnan	33.99 ± 1.10h ^i^	11.68 ± 0.38 ^h^	5.99 ± 0.04 ^h,i^	2.22 ± 0.03 ^a^	6.15 ± 0.26 ^i,j^	20.36 ± 0.31 ^d^	9.43 ± 0.35 ^f^	34.85 ± 0.74 ^c^	Southwest China
Kunming, Yunnan	39.21 ± 1.08 ^e,f^	15.74 ± 0.64 ^f^	ND	1.60 ± 0.06 ^f^	10.18 ± 0.74 ^f^	16.36 ± 0.27 ^i,j^	3.40 ± 0.08 ^o^	19.94 ± 0.52 ^j,k^	
Mean ± SD	36.60 ± 1.09	13.71 ± 0.51	3.00 ± 0.04	1.91 ± 0.04	8.16 ± 0.50	18.36 ± 0.29	6.42 ± 0.21	27.40 ± 0.63	
	**Syringic Acid**	**Vanillin**	***p*-Coumaric Acid + Syringaldehyde**		**Ferulic Acid**	**Sinapic Acid**	**Total**		
Huairou, Beijing	2.80 ± 0.25 ^n,o^	60.11 ± 4.26 ^d^	1.35 ± 0.05 ^n^		3.75 ± 0.18 ^o^	1.25 ^o^	164.43 ± 7.81		North China
Jixian, Tianjin	3.18 ± 0.07 ^m,n^	67.07 ± 0.97 ^b,c^	2.07 ± 0.03 ^l^		11.50 ± 0.18 ^b^	3.57 ± 0.02 ^a^	206.44 ± 2.60		
Tangshan, Hebei	3.52 ± 0.07 ^m^	52.99 ± 2.13 ^e^	1.63 ± 0.02 ^m^		2.84 ± 0.13 ^q^	1.64 ± 0.02 ^l^	159.30 ± 5.79		
Xingtai, Hebei	10.05 ± 0.51 ^a^	59.90 ± 0.93 ^d^	3.18 ± 0.08 ^g,h^		7.78 ± 0.12 ^f^	2.76 ± 0.02 ^e^	208.07 ± 8.31		
Qinhuangdao, Hebei	5.51 ± 0.24 ^f,g,h^	63.45 ± 1.79 ^c,d^	2.42 ± 0.03 ^j,k^		4.50 ± 0.07 ^n^	1.39 ± 0.03 ^m,n^	182.67 ± 3.96		
Shangluo, Shanxi	5.74 ± 0.29 ^e,f,g^	65.28 ± 2.10 ^c^	2.33 ± 0.06 ^k^		4.90 ± 0.26 ^l,m^	2.03 ± 0.06 ^h^	182.21 ± 5.48		Northwest China
Mean ± SD	5.13 ± 0.24	61.47 ± 2.03	2.16 ± 0.04		5.88 ± 0.15	2.11 ± 0.03	183.85 ± 5.66		
Wuxi, Jiangsu	4.80 ± 0.21 ^j,k^	44.26 ± 4.13 ^h^	1.19 ± 0.04 ^n^		3.40 ± 0.13 ^p^	1.07 ± 0.03 ^p^	174.22 ± 8.78		East China
Wenzhou, Zhejiang	5.37 ± 0.29 ^f,g,h,i^	50.51 ± 0.88 ^e,f^	5.76 ± 0.02 ^a^		9.36 ± 0.19 ^c^	2.50 ± 0.06 ^f^	215.72 ± 4.86		
Anqing, Anhui	7.25 ± 0.56 ^d^	49.50 ± 0.64 ^e,f^	4.42 ± 0.07 ^e^		5.40 ± 0.13 ^j,k^	1.33 ^n,o^	237.67 ± 4.38		
Nanping, Fujian	8.91 ± 0.19 ^b^	65.12 ± 2.02 ^c^	3.32 ± 0.14 ^g^		7.35 ± 0.27 ^g^	3.44 ± 0.13 ^b^	203.86 ± 6.59		
Fuzhou, Jiangxi	7.86 ± 0.65 ^c^	70.72 ± 1.34 ^b^	3.58 ± 0.04 ^f^		6.90 ± 0.24 ^h^	1.96 ± 0.06 ^h,i^	314.87 ± 8.71		
Taian, Shandong	5.20 ± 0.27 ^h,i,j^	26.46 ± 1.03 ^j^	3.63 ± 0.17 ^f^		9.15 ± 0.44 ^c,d^	1.85 ± 0.08 ^i,j^	144.28 ± 6.36		
Linyi, Shandong	6.05 ± 0.26 ^e^	45.08 ± 0.76 ^g,h^	3.09 ± 0.08 ^h,i^		6.23 ± 0.16 ^i^	2.85 ± 0.07 ^e^	188.95 ± 3.62		
Mean ± SD	6.49 ± 0.35	50.24 ± 1.54	3.54 ± 0.08		6.83 ± 0.22	2.14 ± 0.06	211.37 ± 6.19		
Xinyang, Henan	4.23 ± 0.43 ^l^	21.23 ± 1.13 ^k^	5.73 ± 0.34 ^a^		5.71 ± 0.26 ^j^	3.55 ± 0.16 ^a,b^	142.35 ± 7.27		Central China
Xiangyang, Hubei	5.84 ± 0.38 ^e,f^	67.00 ± 3.84 ^c^	4.98 ± 0.24 ^c^		8.38 ± 0.25 ^e^	3.00 ± 0.16 ^d^	195.27 ± 9.58		
Mean ± SD	5.03 ± 0.40	44.12 ± 2.49	5.36 ± 0.29		7.04 ± 0.26	3.28 ± 0.16	168.81 ± 8.43		
Yangjiang, Guangdong	5.23 ± 0.22 ^g,h,i,j^	35.88 ± 0.76 ^i^	2.41 ± 0.06 ^j,k^		5.22 ± 0.08 ^k,l^	1.73 ± 0.04 ^k,l^	166.37 ± 4.30		South China
Guilin, Guangxi	4.61 ± 0.21 ^kl,^	61.02 ± 4.11 ^d^	1.69 ± 0.02 ^m^		9.06 ± 0.27 ^c,d^	1.26 ± 0.03 ^o^	186.78 ± 8.94		
Liuzhou, Guangxi	2.43 ± 0.09 ^o^	47.95 ± 2.67 ^f,g^	2.91 ± 0.08 ^i^		4.82 ± 0.21 ^m,n^	1.48 ± 0.05 ^m^	161.28 ± 10.13		
Haikou, Hainan	6.78 ± 0.25 ^d^	99.33 ± 1.50 ^a^	2.57 ± 0.06 ^j^		13.27 ± 0.25 ^a^	1.76 ± 0.05 ^j,k^	240.43 ± 5.04		
Mean ± SD	4.76 ± 0.19	61.05 ± 2.26	2.40 ± 0.05		8.09 ± 0.20	1.56 ± 0.04	188.72 ± 7.10		
Zhaotong, Yunnan	4.87 ± 0.40 ^i,j,k^	52.08 ± 1.59 ^e^	4.77 ± 0.11 ^d^		8.84 ± 0.09 ^d^	3.16 ± 0.03 ^c^	198.37 ± 5.42		Southwest China
Kunming, Yunnan	3.31 ± 0.15 ^m,n^	27.31 ± 1.23 ^j^	5.36 ± 0.16 ^b^		4.77 ± 0.07 ^m,n^	2.30 ± 0.03 ^g^	154.11 ± 5.03		
Mean ± SD	4.09 ± 0.28	39.70 ± 1.41	5.06 ± 0.13		6.80 ± 0.08	2.73 ± 0.03	176.24 ± 5.23		

Data were expressed as mean ± standard deviation (*n* = 3). Data in the same column marked with different superscript letters (^a–p^) differed significantly (*p* ≤ 0.05).

**Table 6:** Results of phenolic profiles and antioxidant values based on morphological properties.

Mean	TPC (mg GAE/g)	TFC (mg CAE/g)	CTC (mg CAE/g)	DPPH (μmol TE/g)	FRAP (mM FE/100 g)	ABTS (μmol TE/g)
*Banli*	1.76 ± 0.33 ^a^	0.82 ± 0.17 ^a^	9.75 ± 3.05 ^a^	10.84 ± 1.89 ^a^	2.04 ± 0.95 ^a^	15.57 ± 3.63 ^a^
*Maoli*	1.61 ± 0.44 ^a,b^	0.77 ± 0.14 ^a,b^	7.67 ± 3.31 ^b^	10.51 ± 3.26 ^a,b^	1.73 ± 0.94 ^b^	14.25 ± 6.13 ^a^
*Youli*	1.47 ± 0.24 ^b^	0.70 ± 0.16 ^b^	6.14 ± 2.69 ^b^	9.11 ± 1.27 ^b^	1.47 ± 0.33 ^c^	11.27 ± 1.25 ^b^

Values are expressed as the mean of triplicates ± standard deviation. Means in the same column with unlike superscripts (^a–c^) differ significantly (*p* < 0.05).

**Table 7:** Correlation analysis between phenolic contents, antioxidant activities, and color value.

Correlation Coefficient (r)		TPC	TFC	CTC	DPPH	FRAP	ABTS
TPC		-	-	-	-	-	-
TFC		0.762 **	-	-	-	-	-
CTC		0.834 **	0.708 **	-	-	-	-
DPPH		0.821 **	0.575 **	0.782 **	-	-	-
FRAP		0.866 **	0.540 *	0.719 **	0.819 **	-	-
ABTS		0.884 **	0.684 **	0.866 **	0.875 **	0.819 **	-
Color	L	−0.214	−0.114	−0.182	−0.444 *	−0.162	−0.374
	a	0.539 *	0.515 *	0.483 *	0.471 *	0.423	0.539 *
	b	−0.344	−0.502 *	−0.363	−0.180	−0.167	−0.209

(Sample size: *N* = 21, *p* < 0.05 was recorded as *; *p* < 0.01 was recorded as **).

**Table 8:** Phenolic acid contents of chestnut based on morphological features.

Variety	Gallic Acid	Protocatechuic Acid	2,3,4-Trihydroxybenzoic Acid	Protocatechualdehyde	*p*-Hydroxybenzoic Acid	Gentisic Acid	Chlorogenic Acid	Vanillic Acid + Caffeic Acid
*Youli*	41.30 ± 1.34 ^a^	20.47 ± 0.78 ^a^	5.11 ± 0.10 ^b^	1.75 ± 0.07 ^a^	10.66 ± 0.72 ^a^	18.98 ± 0.61 ^a^	8.60 ± 0.33 ^a,b^	26.06 ± 0.73 ^a^
*Banli*	38.14 ± 1.08 ^ab^	16.59 ± 0.74 ^b^	4.27 ± 0.07 ^c^	1.71 ± 0.04 ^a^	8.55 ± 0.39 ^b^	15.11 ± 0.64 ^b^	9.59 ± 0.27 ^a^	24.24 ± 0.61 ^a^
*Maoli*	34.41 ± 1.00 ^b^	12.59 ± 0.45 ^c^	5.29 ± 0.05 ^a^	1.43 ± 0.03 ^b^	7.83 ± 0.44 ^b^	15.72 ± 0.50 ^b^	7.67 ± 0.21 ^b^	23.47 ± 0.57 ^a^
**Variety**	**Syringic Acid**		**Vanillin**	***p*-Coumaric Acid + Syringaldehyde**	**Ferulic Acid**	**Sinapic Acid**	**Total Phenolic Content**	
*Youli*	5.22 ± 0.40 ^a^		53.91 ± 2.29 ^a^	4.28 ± 0.14 ^a^	6.82 ± 0.22 ^a^	2.45 ± 0.09 ^a^	206.53 ± 7.65 ^a^	
*Banli*	5.63 ± 0.24 ^a^		55.85 ± 1.70 ^a^	2.95 ± 0.08 ^b^	6.73 ± 0.17 ^a^	2.22 ± 0.04 ^a^	193.43 ± 6.03 ^a,b^	
*Maoli*	5.19 ± 0.27 ^a^		50.69 ± 1.90 ^a^	2.87 ± 0.07 ^b^	6.95 ± 0.20 ^a^	1.90 ± 0.04 ^b^	176.78 ± 5.74 ^b^	

Values are expressed as the mean ± standard deviation. Means in the same column with unlike superscripts (^a–c^) differ significantly (*p* < 0.05).

## Supplementary Materials

The following are available online at https://www.mdpi.com/2076-3921/9/3/190/s1, Table S1: The maximum detection wavelengths, retention time, regressive equations and correlation coefficient of 14 phenolic acids, Figure S1: Chestnut sampling regions in China (marked by stars), Figure S2: Twenty one raw chestnut samples investigated in this study. Figure S3: Chromatogram of 14 phenolic acids. Figure S4: Correlation coefficient (r) between temperature and sunlight with phenolic contents and antioxidant activities.
