# Supplementary material for: Comparative Study on Phytochemical Profiles and Antioxidant Capacities of Chestnuts Produced in Different Geographic Area in China
Source: Antioxidants (Basel). 2020 Feb 25;9(3):190. doi: 10.3390/antiox9030190 (PMC7139719; doi:10.3390/antiox9030190)
Supplement: Supplementary file 1 [file antioxidants-09-00190-s001.pdf]

Supplemental Table 1. The maximum detection wavelengths, retention time, regressive equations and correlation coefficient of 14 phenolic acids

Code	Name	Detection wavelength	Retention time (min)	Regression equation	R^2
1	Gallic acid	220 nm	8.95	y = 226998x	0.9994
2	Protocatechuic acid	220 nm	15.86	y = 188665x + 3486.9	0.9997
3	2,3,4-Trihydroxybenzoic acid	260 nm	18.14	y = 142439x - 131628	0.9994
4	Protocatechualdehyde	230 nm	21.97	y = 158161x - 7692	0.9998
5	*p*-Hydroxybenzoic acid	260 nm	24.55	y = 229679x + 419.61	0.9998
6	Gentisic acid	210 nm	27.29	y = 317082x – 4429.3	0.9997
7	Chlorogenic acid	320 nm	31.55	y = 111727x – 6976.3	0.9998
8	Vanillic acid + Caffeic acid	220 nm	32.90	y = 366259x + 8460.1	0.9998
9	Syringic acid	220 nm	38.69	y = 322482x + 18809	0.9997
10	Vanillin	230 nm	39.57	y = 6538.8x – 3890.8	0.9998
11	*p*-Coumaric acid + Syringaldehyde	320 nm	45.11	y = 327787x - 47265	0.9998
12	Ferulic acid	320 nm	51.60	y = 168386x - 22016	0.9998
13	Sinapic acid	320 nm	54.47	y = 214299x - 29985	0.9997
14	Salicylic acid	240 nm	64.29	y = 132746x - 23350	0.9997

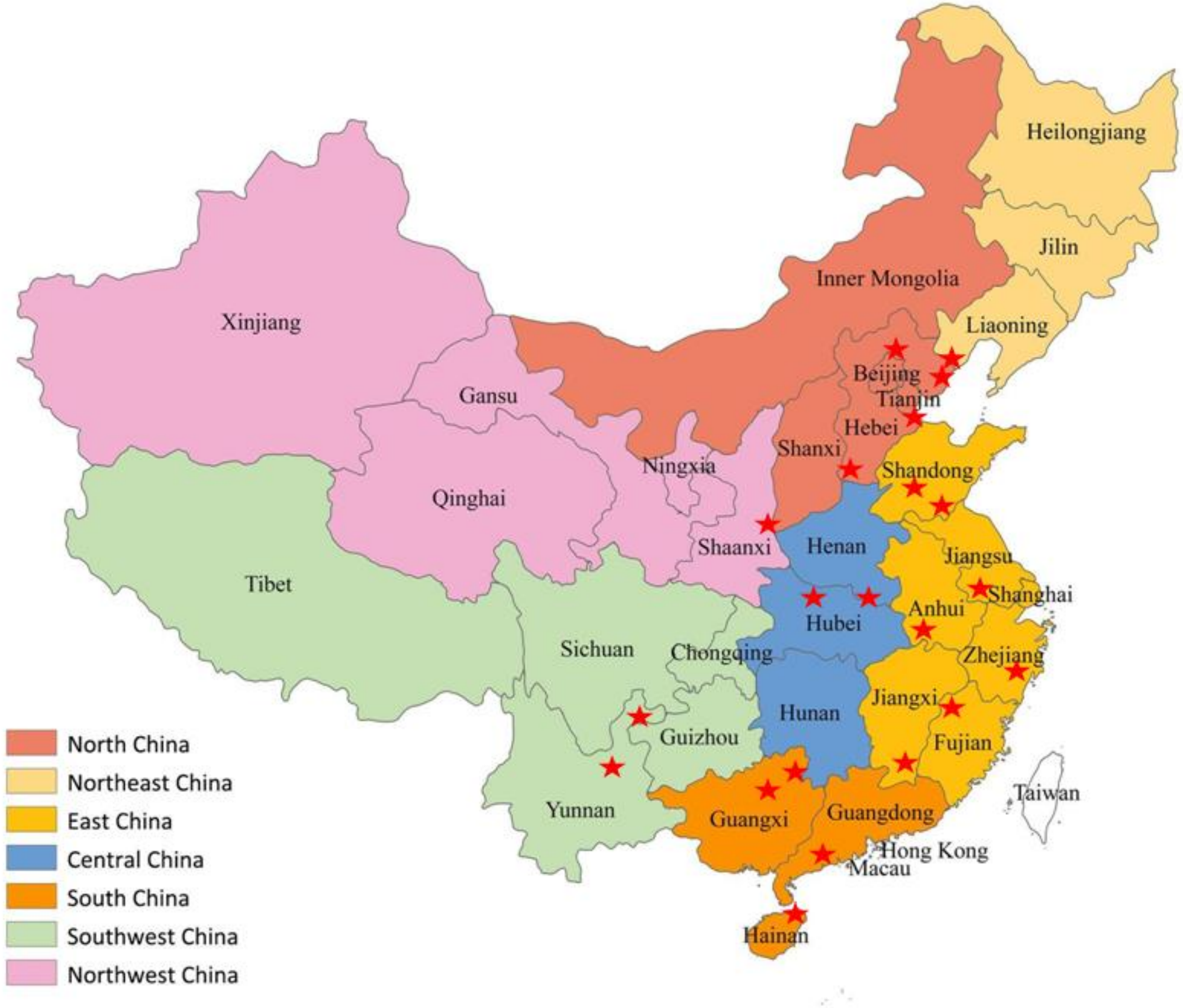

Supplemental Fig 1. Chestnut sampling regions in China (marked by stars)

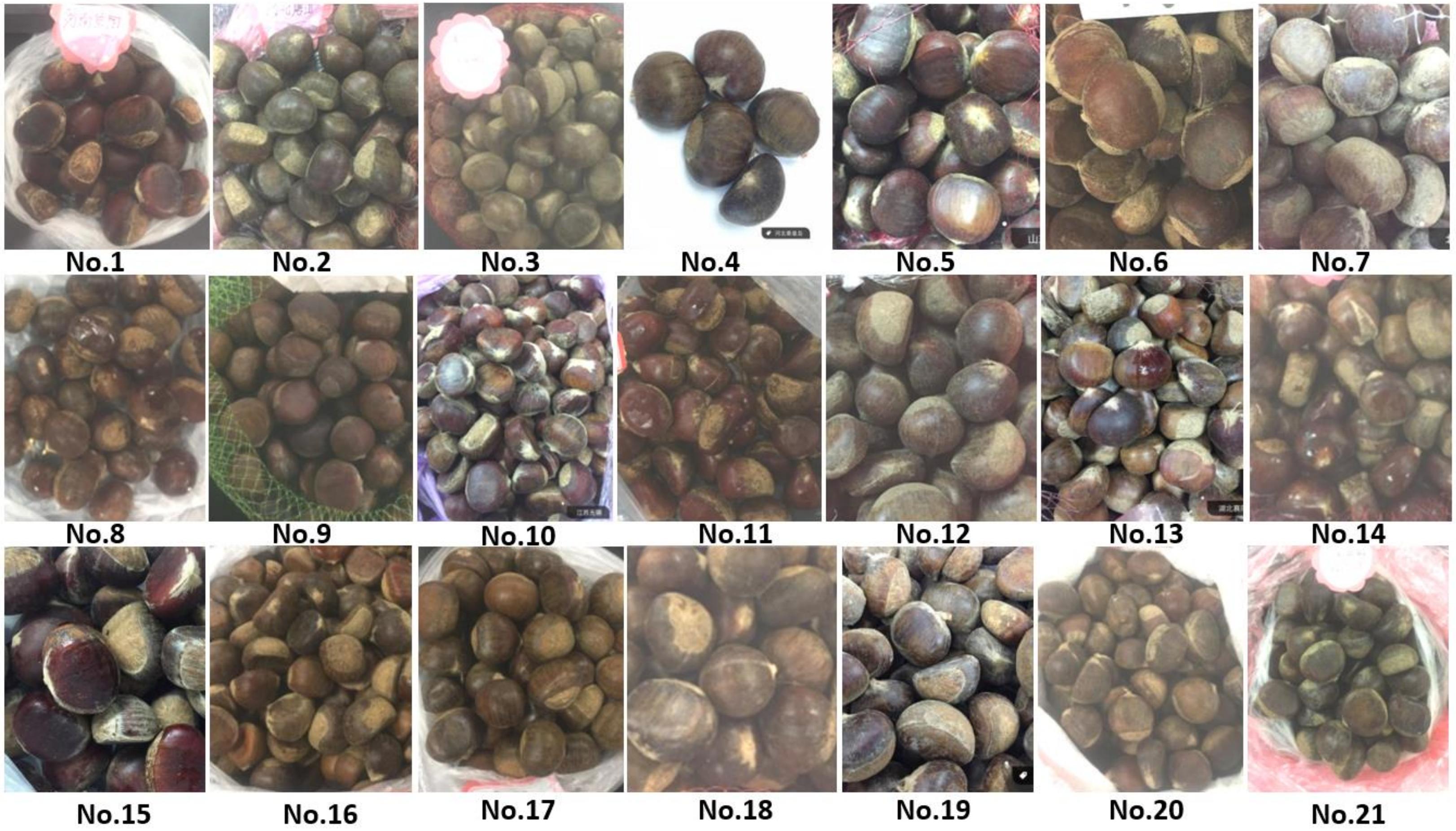

Supplemental Fig. 2. Twenty one raw chestnut samples investigated in this study.

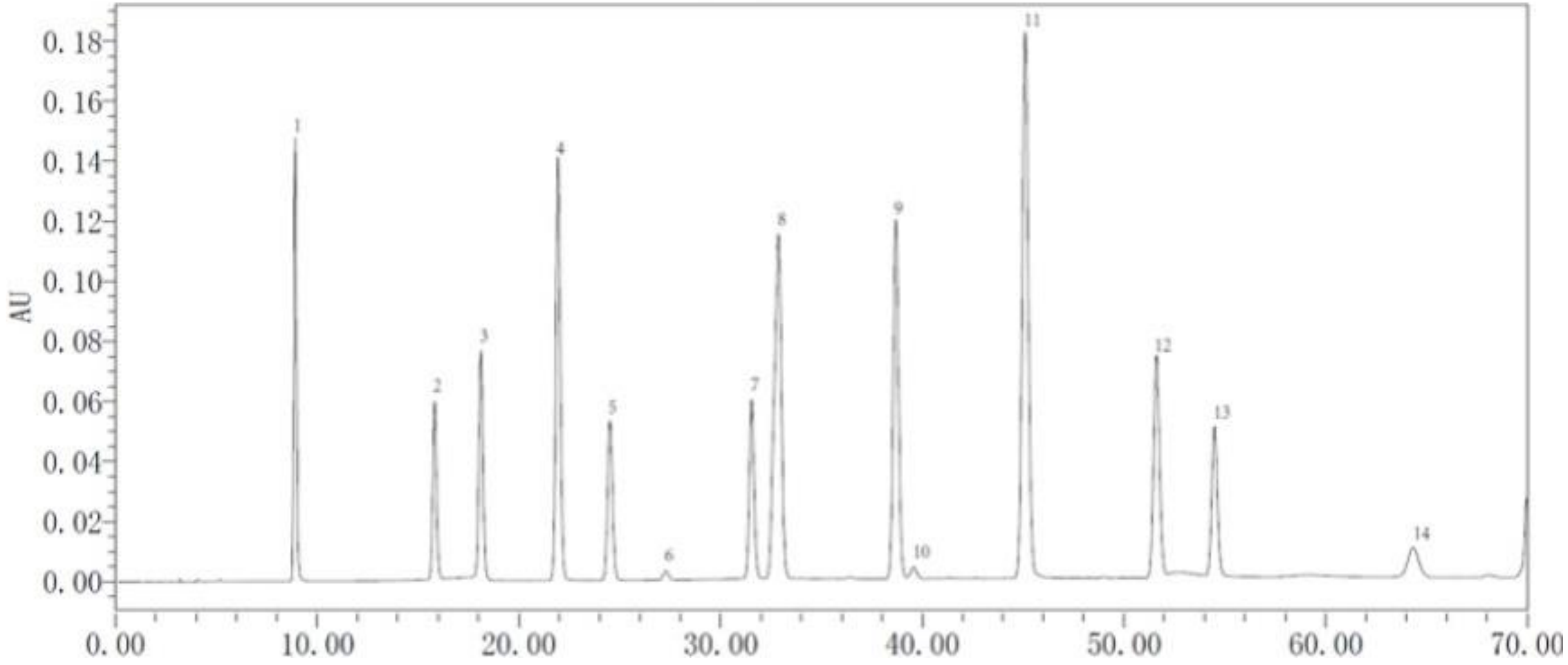

Supplemental Fig. 3. Chromatogram of 14 phenolic acids.

1: Gallic acid; 2: Protocatechuic acid; 3: 2,3,4-trihydroxybenzoic acid; 4: Protocatechualdehyde; 5: p-hydroxybenzoic acid; 6: Gentisic acid; 7: Chlorogenic acid; 8: Vanillic acid+Caffeic acid; 9: Syringic acid; 10: Vanillin; 11: p-coumaric acid+Syringaldehyde; 12: Ferulic acid; 13: Sinapic acid; 14: Salicylic acid.

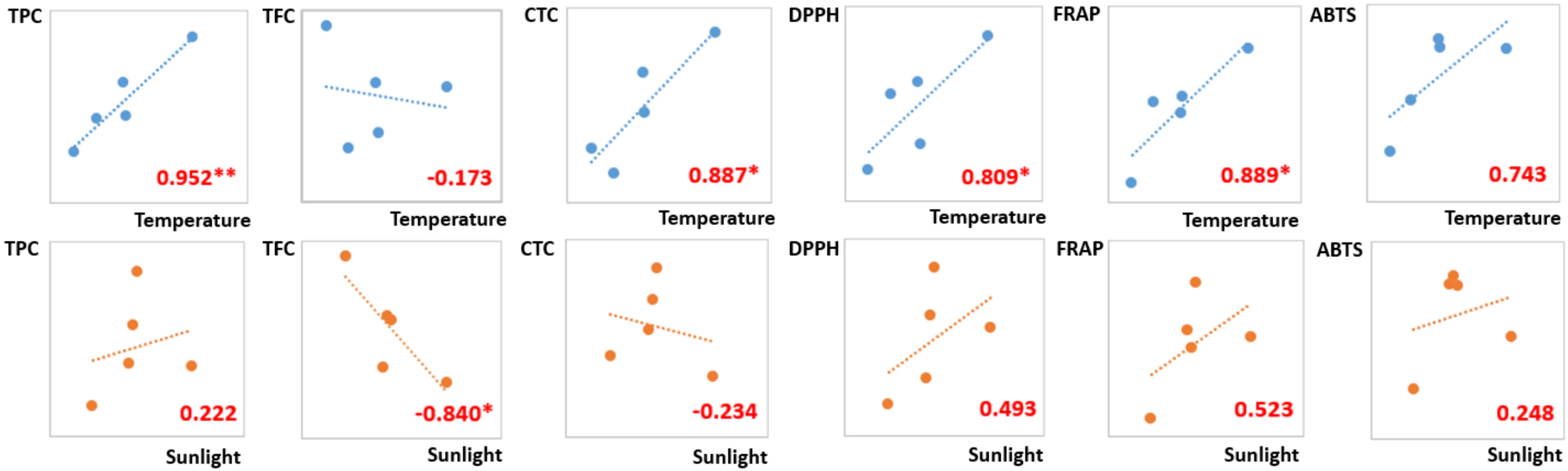

Supplemental Fig 4. Correlation coefficient (r) between temperature and sunlight with phenolic contents and antioxidant activities
